# 67863 Insulin use and depigmentation: a survey of real-world evidence

**DOI:** 10.1017/cts.2021.484

**Published:** 2021-03-31

**Authors:** James Lukasik, Zachary A.P. Wintrob, G. Emilia Costin, Alice C. Ceacareanu

**Affiliations:** 1 Hartwick College; 2 ROAKETIN Inc.; 3 Institute for In Vitro Sciences

## Abstract

ABSTRACT IMPACT: Long-acting insulin containing protamine is more likely to be associated with skin depigmentation. OBJECTIVES/GOALS: An acquired disorder, skin depigmentation was found to be significantly correlated with diabetes. While a recent meta-analysis pointed at a possible similar pathogenesis, the possibility of vitiligo occurring as a drug-induced disease was never explored. This study aimed at elucidating whether utilization of specific insulins may play a role. METHODS/STUDY POPULATION: Records from the Medical Panels Expenditure Survey (MEPS) database made available by the Agency for Healthcare Research and Quality were used to identify all injectable insulin users (n=8867). ICD-9/10 codes were abstracted from the medical conditions files for all the subjects reporting any type of injectable insulin use (1996-2017). Skin depigmentation codes identified in our dataset were 709 and L81. Insulins were categorized based on duration of action, short-acting (SA), intermediate-acting (IA), and long-acting (LA), as well as based on formulation ingredients (zinc, protamine-zinc, other), and insulin combination (SA with or without IA/LA containing or not protamine-zinc). The association between skin depigmentation occurrence and insulin type and/or category was assessed by Fisher’s exact test. RESULTS/ANTICIPATED RESULTS: A total of 225 out of 8867 individuals were diagnosed with skin depigmentation. Incidence of skin depigmentation was 2.25% in SA users (n1=3606, p=0.355), 2.24% in LA users (n2=3910, p=0.337), and 2.39% in IA users (n3=4015, p=0.062). Occurrence of skin depigmentation was similar between users of insulin mono- or combo therapy (p=0.758). Interestingly, among IA insulins, insulin protamine-zinc insulin (n4=3992) distinguished as being mainly responsible for the association with the occurrence of skin depigmentation (p=0.062), whereas insulin zinc was not (n5=37, p=1.000). The highest skin depigmentation incidence was observed among Pacific Islanders (2.66%, p=0.110). Males distinguished by a skin depigmentation incidence of 2.34% vs. 1.91% in females (p=0.086). DISCUSSION/SIGNIFICANCE OF FINDINGS: We report that presence of protamine-zinc may play a role in the development of skin depigmentation. It is uncertain whether this risk may be shared equally by insulin users diagnosed with type 1 and type 2 diabetes. Of note, we observed a higher skin depigmentation incidence than that reported by community- (0.2%) or hospital-based (1.8%) studies.

